# Differing association of alcohol consumption with different stroke types: a systematic review and meta-analysis

**DOI:** 10.1186/s12916-016-0721-4

**Published:** 2016-11-24

**Authors:** Susanna C. Larsson, Alice Wallin, Alicja Wolk, Hugh S. Markus

**Affiliations:** 1 Unit of Nutritional Epidemiology, Institute of Environmental Medicine, Karolinska Institutet, SE-17177 Stockholm, Sweden; 2 Stroke Research Group, Neurology Unit, Department of Clinical Neurosciences, University of Cambridge, Cambridge, CB2 0QQ UK

**Keywords:** Alcohol consumption, Meta-analysis, Prospective studies, Stroke

## Abstract

**Background:**

Whether light-to-moderate alcohol consumption is protective against stroke, and whether any association differs by stroke type, is controversial. We conducted a meta-analysis to summarize the evidence from prospective studies on alcohol drinking and stroke types.

**Methods:**

Studies were identified by searching PubMed to September 1, 2016, and reference lists of retrieved articles. Additional data from 73,587 Swedish adults in two prospective studies were included. Study-specific results were combined in a random-effects model.

**Results:**

The meta-analysis included 27 prospective studies with data on ischemic stroke (25 studies), intracerebral hemorrhage (11 studies), and/or subarachnoid hemorrhage (11 studies). Light and moderate alcohol consumption was associated with a lower risk of ischemic stroke, whereas high and heavy drinking was associated with an increased risk; the overall RRs were 0.90 (95 % CI, 0.85–0.95) for less than 1 drink/day, 0.92 (95 % CI, 0.87–0.97) for 1–2 drinks/day, 1.08 (95 % CI, 1.01–1.15) for more than 2–4 drinks/day, and 1.14 (95 % CI, 1.02–1.28) for more than 4 drinks/day. Light and moderate alcohol drinking was not associated with any hemorrhagic stroke subtype. High alcohol consumption (>2–4 drinks/day) was associated with a non-significant increased risk of both hemorrhagic stroke subtypes, and the relative risk for heavy drinking (>4 drinks/day) were 1.67 (95 % CI, 1.25–2.23) for intracerebral hemorrhage and 1.82 (95 % CI, 1.18–2.82) for subarachnoid hemorrhage.

**Conclusion:**

Light and moderate alcohol consumption was inversely associated only with ischemic stroke, whereas heavy drinking was associated with increased risk of all stroke types with a stronger association for hemorrhagic strokes.

**Electronic supplementary material:**

The online version of this article (doi:10.1186/s12916-016-0721-4) contains supplementary material, which is available to authorized users.

## Background

Whether light-to-moderate alcohol consumption, generally defined as 1–2 drinks per day, is protective against cardiovascular disease remains a controversial topic. Alcohol consumption in moderation has been associated with increased high-density lipoprotein cholesterol, improved insulin sensitivity, and decreased levels of fibrinogen and inflammatory markers [1–4]. Moreover, alcohol intake is associated with risk of hypertension in a linear positive dose–response relationship in men and with a J-shaped dose–response relationship in women [5]. Whereas observational studies have consistently reported an inverse association between moderate alcohol consumption and risk of ischemic heart disease [6, 7], the association between alcohol consumption and risk of stroke types has not been delineated. Previous meta-analyses of alcohol consumption and stroke risk examined only total stroke (ischemic and all hemorrhagic strokes combined) [6] or combined case-control and prospective studies in the analysis of stroke types [8, 9]. Whether the association of alcohol consumption with risk of hemorrhagic stroke varies for intracerebral hemorrhage and subarachnoid hemorrhage was not addressed in previous meta-analyses.

The aim of this study was to perform a contemporary systematic review and meta-analysis to summarize available evidence from prospective studies on alcohol consumption in relation to risk of ischemic stroke, intracerebral hemorrhage, and subarachnoid hemorrhage. Moreover, we investigated the association between alcohol consumption and incidence of stroke types in 4555 stroke cases with a mean of 11.9 years of follow-up in the Cohort of Swedish Men (COSM) and the Swedish Mammography Cohort (SMC), and included these cohorts in the present meta-analysis.

## Methods

### Swedish cohort studies

Details about the COSM and the SMC, assessment of alcohol consumption and covariates, case ascertainment, and statistical analysis are reported in Additional file 1: Text S1. The study population for this analysis consisted of 39,941 men (45–79 years of age) in the COSM and 33,646 women (49–83 years of age) in the SMC. Participants were free of stroke and ischemic heart disease at baseline (January 1, 1998) and were followed up through December 31, 2010.

### Meta-analysis

#### Search strategy

The design, analysis, and reporting for this meta-analysis followed the MOOSE guidelines [10]. Relevant studies were identified by a literature search of the PubMed database (from January 1966 to September 1, 2016), without restrictions, using the search terms “alcohol consumption”, “alcohol drinking”, or “alcohol intake” combined with “stroke”, or “cerebrovascular disease”, or “cerebral infarction”, or “intracerebral hemorrhage” or “subarachnoid hemorrhage”. The database search was performed by two authors (SCL and AWa) and enhanced by searches of the reference lists of identified articles.

#### Inclusion criteria

Two authors (SCL and AWa) independently evaluated all records by title, abstract, or full text for potentially eligible studies, and any disagreement was resolved by consensus. Eligible for inclusion in the meta-analysis were prospective studies that reported relative risks (RR) with 95 % confidence intervals (CI) for quantitative categories of alcohol consumption in relation to non-fatal or fatal ischemic stroke, intracerebral hemorrhage, or subarachnoid hemorrhage. Studies that only reported data on total stroke (ischemic and hemorrhagic strokes combined) or total hemorrhagic stroke were not eligible. Where duplicate publications were available from the same study population, the study with data on stroke types and the largest number of stroke cases was included.

#### Data extraction and quality assessment

Details recorded for each study were the first author’s surname, publication year, study name, country in which the study was performed, number of subjects, proportion of men, age range, mean follow-up, number of cases for each stroke type, method used for assessment of alcohol consumption, average or range of alcohol intake as well as number of cases and person-years (or non-cases if person-years were not provided) in each category, RRs with 95 % confidence intervals (CIs) for each category, and covariates controlled for in the most fully adjusted multivariable model. Study quality was evaluated with the Newcastle–Ottawa Scale [11]. The quality score ranged from 0 to 9. Details of how the criteria were applied are described in Additional file 1: Figure S1.

#### Statistical analysis

To place the studies on a common scale, alcohol consumption was standardized to drinks of alcohol. If alcohol consumption was reported in grams, the values were converted into drinks by assuming that one drink on average contains 12 grams of alcohol. The median or mean alcohol intake for each category was assigned to the corresponding risk estimate. If average values were not reported, each category was assigned the midpoint of the upper and lower boundaries for that category. If an upper boundary was not provided for the highest category, the boundary was presumed to have the same range as the adjacent category. In a sensitivity analysis, for studies that did not provide the average value for the highest category, the mid-point for the highest category was set at 1.5 times the half range of the preceding category.

A random-effects model was used to combine study-specific RRs by alcohol consumption categories based on the average consumption in each category. The exposure categories were as follows: light (< 1 drink/day), moderate (1–2 drinks/day), high (> 2–4 drinks/day), and heavy (> 4 drinks/day) alcohol consumption. The comparison group was the reference group in each study (i.e., non-drinkers, never drinkers, or occasional drinkers). In a sensitivity analysis, we stratified the studies by reference group used.

Meta-regression and subgroup analyses were performed to assess whether the association between alcohol consumption and stroke types varied by geographic region (United States, Europe, and Asia), sex, and study quality (Newcastle–Ottawa Scale: < 7 vs. ≥ 7). Because of the small number of studies in some subgroups, the light and moderate alcohol consumption categories and the high and heavy alcohol consumption categories were combined. In a sensitivity analysis for women, we defined 1 or less drinks/day as the moderate alcohol consumption category and more than 1 drink/day as high-to-heavy alcohol drinking. Between-study heterogeneity was evaluated with the *I* ^*2*^ statistic [12], and the degree of heterogeneity was quantified using the following cutoff values: less than 30 %, no or low heterogeneity; 30–75 %, moderate heterogeneity; and more than 75 %, notable heterogeneity. Egger’s test was used to assess small-study bias such as publication bias [13]. Statistical tests were considered statistically significant at *P* values < 0.05. All analyses were conducted using Stata (version 14.1, StataCorp, College Station, TX).

## Results

### Swedish cohort studies

Baseline characteristics of men in the COSM and women in the SMC are shown in Additional file 1: Table S1. A total of 3824 ischemic stroke cases (2216 in men and 1608 in women), 555 intracerebral hemorrhage cases (350 in men and 205 in women), and 176 subarachnoid hemorrhage cases (82 in men and 94 in women) were ascertained over 873,440 person-years (mean 11.9 years) of follow-up. There was no statistically significant association between alcohol consumption and risk of ischemic stroke (Additional file 1: Table S2). However, because of the small number of cases in some categories, we cannot exclude weak associations. Consumption of more than 21 drinks/week of alcohol was associated with a statistically significant increased risk of intracerebral hemorrhage in both men and women (Additional file 1: Table S2). Alcohol consumption was statistically significantly positively associated with subarachnoid hemorrhage in women but not in men (Additional file 1: Table S2).

### Meta-analysis

We screened 2505 abstracts or titles and reviewed 89 full-text articles (Additional file 1: Figure S2). A total of 27 prospective studies (29 publications [14–42]), including the COSM and the SMC, on alcohol consumption in relation to risk of one or more stroke types were included in the meta-analysis. Combined, these studies included 19,302 ischemic stroke cases (25 studies), 2359 intracerebral hemorrhage cases (11 studies), and 1164 subarachnoid hemorrhage cases (11 studies). Characteristics of the included studies are shown in Table 1. Of the 27 studies, ten were conducted in Europe (four in Sweden, two in Finland and one each in Denmark, Norway, the Netherlands, and Germany), nine in the United States, and eight in Asia. Alcohol consumption was assessed with a self-administered questionnaire in all but six studies [14, 16, 17, 19, 28, 29] in which alcohol consumption was assessed by an interview. Most studies adjusted for major potential confounders such as age, sex, smoking, body mass index, and diabetes mellitus (Table 1).

**Table 1:** Prospective studies of alcohol consumption and risk of ischemic stroke, intracerebral hemorrhage, and subarachnoid hemorrhage

						No. of stroke cases				
Study	Cohort name, country	No. of subjects	Age, years	Men, %	Follow-up, years	IS	ICH	SAH	NOS score	Adjustments
Donahue et al., 1986 [14]	Honolulu Heart Program, USA	8006	45–69	100	12	190	44	32	8	Age, smoking, BMI, hypertensive status, serum cholesterol, uric acid glucose, and hematocrit concentrations
Stampfer et al., 1988 [15]	Nurses’ Health Study, USA	87,526	34–59	0	3.8	–^a^	–	28	7	Age, smoking, obesity, exercise, family history of myocardial infarction, menopausal status and hormone use, history of hypertension, high cholesterol levels, diabetes, intake of cholesterol, saturated fat, and polyunsaturated fat
Iso et al., 1995 [16]	Rural Japanese Cohorts, Japan	2890	40–69	100	10.5	104	–	–	8	Age, smoking, hypertension, serum total cholesterol, and diabetes
Kiyohara et al., 1995 [17]	The Hisayama Study, Japan	1621	≥ 40	44	26	244	60	–	9	Age, sex, BMI, hypertension, electrocardiographic abnormalities, heart rate, glucose intolerance, serum cholesterol, and smoking
Leppälä et al., 1999 [18]	Alpha-Tocopherol, Beta-Carotene Cancer Prevention Study, Finland	26,556	50–69	100	6.1	733	95	83	7	Age, randomized treatment assignment, education, smoking, BMI, physical activity, serum total cholesterol, history of heart disease, diabetes
Sankai et al., 2000 [19]	Six Japanese Communities, Japan	12,372	40–69	40	9.4	–	–	71	8	Age, sex, smoking, BMI, blood pressure, serum total cholesterol, diabetes
Suh et al., 2001 [20]	Korea Medical Insurance Corporation Study, Korea	114,793	35–59	100	5.4	–	373	98	6	Age, smoking, BMI, blood pressure, fasting blood glucose, total serum cholesterol
Klatsky et al., 2001 [21]	Kaiser Permanente Medical Care Program Cohort, USA	128,934	< 40–70+	44	18	2014	–	–	6	Age, sex, race, education, smoking, BMI
Klatsky et al., 2002 [22]	Kaiser Permanente Medical Care Program Cohort, USA	128,934	< 40–70+	44	18	–	299	133	6	Age, sex, race, education, smoking, BMI
Djousse et al., 2002 [23]	Framingham Study, USA	9171	≥ 50	42	8.8	441	–	–	7	Age, smoking, BMI, diabetes
Iso et al., 2004 [24]	Japan Public Health Centre-Based Prospective Study, Japan	19,356	40–59	100	11.0	319	219	73	6	Age, education, public health centers, smoking, BMI, sports at leisure time, history of diabetes, intakes of fruit, vegetable, and fish
Iwashita et al., 2005 [25]	Kyushu Lipid Intervention Study, Japan	4349	45–74	100	5	81	–	–	6	Age, smoking, BMI, serum total cholesterol, high-density lipoprotein cholesterol, prior use of cholesterol-lowering drugs, history of angina, hypertension, and diabetes
Mukamal et al, 2005 [26]	Cardiovascular Health Study, USA	4410	≥ 65	36	9.2	434	–	–	7	Age, sex, geographic region, parental history of myocardial infarction, smoking, BMI, physical activity, hypercholesterolemia, diabetes, aspirin use, intakes of energy, vitamin E, folate, saturated fat, trans fats, omega-3 fatty acids, potassium, magnesium, and dietary fiber
Nielsen et al., 2005 [27]	Copenhagen City Heart Study, Denmark	12,096	21–98	44	15	786	–	–	8	Age, sex, education, smoking, BMI, physical activity, systolic blood pressure, history of myocardial infarction and diabetes, Forced Expiratory Volume 1
Elkind et al., 2006 [28]	Northern Manhattan Study, USA	3176	≥ 40	37	5.9	172	–	–	8	Age, sex, race/ethnicity, education, smoking, high-density lipoprotein cholesterol, history of hypertension, atrial fibrillation, and diabetes
Bazzano et al., 2007 [29]	China National Hypertension Survey Epidemiology Follow-up Study, China	64,338	≥ 40	100	7.7	1724	–	–	8	Age, geographic region, urban or rural residence, education, smoking, BMI, physical activity, diabetes
Sturgeon et al, 2007 [30]	Atherosclerosis Risk in Communities Study and Cardiovascular Health Study, USA	21,680	≥ 45	45 and 42	12.2	–	135	–	5	Age
Chiuve et al., 2008 [31]^b^	Health Professionals Follow-up Study, USA	43,685	40–75	100	18	600	–	–	6	Age, calendar year, family history of myocardial infarction, aspirin use, vitamin E supplementation
Lu et al., 2008 [32]	Swedish Women’s Lifestyle and Health Cohort Study, Sweden	45,449	30–50	0	11	111	47	–	7	Age, education, BMI, smoking, parity and age at first birth, oral contraceptive use
Sandvei et al., 2009 [33]	Nord-Trøndelag Health Study, Norway	61,371	≥ 20	49	22	–	–	132	4	Age, sex
Bos et al., 2010 [34]	European Investigation into Cancer and Nutrition-Netherlands Cohort, The Netherlands	10,530	20–70	0	9.4	165	–	–	8	Age, cohort, education, smoking, BMI, physical activity, menopausal status, hypercholesterolemia, diabetes, antihypertensive medication, intakes of energy, vitamin E, vitamin C, saturated fat, and fiber
Rist et al., 2010 [35]	Physicians’ Health Study, USA	21,860	40–84	100	21.6	1157	–	–	8	Age, smoking, BMI, exercise, systolic blood pressure, treatment for hypertension, diabetes, and migraine
Zhang et al., 2011 [36]	Six geographic areas of Finland	36,686	25–74	47	13.7	1167	–	–	8	Age, study year, sex, education, smoking, BMI, physical activity, systolic blood pressure, total cholesterol level, family history of stroke, diabetes, intake of fruit and vegetables
Higashiyama et al., 2011 [37]	Suita Study, Japan	2336	30–79	100	12.5	78	–	–	7	Age, smoking, BMI, high-density lipoprotein cholesterol, triglycerides, history of hypercholesterolemia, hypertension, and diabetes
Drogan et al., 2012 [38]	European Investigation into Cancer and Nutrition-Potsdam cohort, Germany	2175	35–65	37	8.2	169	–	–	7	Age, education, smoking, BMI, waist circumference, physical activity, hypertension, diabetes, plasma total cholesterol levels, and non-alcohol energy intake
Jimenez et al., 2012 [39]	Nurses’ Health Study, USA	83,578	30–55	0	20.3	1206	–	–	8	Age, education, marital status, family history of heart disease, smoking, BMI, physical activity, postmenopausal status, hormone therapy use, aspirin use, high cholesterol, history of heart disease and diabetes, bilateral oophorectomy, multivitamin use, diet score
Ikehara et al., 2013 [40]	Japan Public Health Centre-Based Prospective Study, Japan	47,100	40–69	0	16.7	964	532	338	8	Age, area, smoking, BMI, sports at leisure-time, history of hypertension and diabetes, flushing after alcohol drinking, mental stress, menopausal status
Kadlecová et al., 2015 [41]	Swedish Twin Registry, Sweden	11,644	≤ 60	30	29.1	1846	–	–	8	Age, sex, smoking, BMI, exercise, history of coronary heart disease, hypertension, diabetes and depression, stress reactivity
Jones et al., 2015 [42]	Atherosclerosis Risk in Communities study, USA	12,433	45–64	45	22.6	773	–^c^	–	6	Age, sex, center-race interaction, education, smoking; IS analyses were further adjusted for low-density lipoprotein cholesterol, history of coronary heart disease and diabetes, marital status, diet score
Larsson et al., 2016 (current study)	Cohort of Swedish Men, Sweden	39,941	45–79	100	11.9	2216	350	82	8	Age, education, family history of myocardial infarction, smoking, BMI, exercise, walking/bicycling, history of hypertension, hypercholesterolemia, atrial fibrillation, and diabetes, aspirin use, diet score
Larsson et al., 2016 (current study)	Swedish Mammography Cohort, Sweden	33,646	49–83	0	11.9	1608	205	94	8	Age, education, family history of myocardial infarction, smoking, BMI, exercise, walking/bicycling, history of hypertension, hypercholesterolemia, atrial fibrillation, and diabetes, aspirin use, diet score

*BMI* body mass index, *HS* hemorrhagic stroke, *ICH* intracerebral hemorrhage, *IS* ischemic stroke, *NOS* Newcastle–Ottawa Scale, *SAH* subarachnoid hemorrhage

^a^This study also reported results for ischemic stroke, but results for this stroke type were later reported in another study with longer follow-up [39]

^b^This study also reported results from the Nurses’ Health Study, but results from this cohort were later reported in another study with longer follow-up [39]

^c^This study also reported results for intracerebral hemorrhage, but results for this stroke type were also reported in another article [30] that combined data from this cohort with those from another cohort and included a larger number of intracerebral hemorrhage cases

#### Overall analyses

**Fig. 1:**
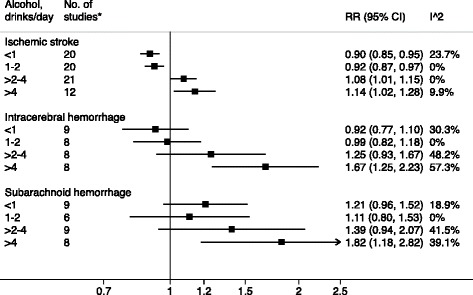
Overall relative risks (RR) with 95 % confidence intervals (CI) for the associations of alcohol consumption (average number of drinks per day) with risk of ischemic stroke, intracerebral hemorrhage, and subarachnoid hemorrhage. *Number of studies that contributed data to each category of alcohol consumption and stroke type. *I* ^*2*^ values < 30 %, 30–75 %, and > 75 % were interpreted as no or low heterogeneity, moderate heterogeneity, and notable heterogeneity, respectively

The associations between levels of alcohol consumption and stroke types are presented in Fig. 1. Light and moderate alcohol consumption (up to 2 drinks/day) was associated with a reduced risk of ischemic stroke, whereas high and heavy alcohol consumption (> 2 drinks/day) was associated with an increased risk. The overall RRs (95 % CI) of ischemic stroke were 0.90 (95 % CI, 0.85–0.95) for less than 1 drink/day, 0.92 (95 % CI, 0.87–0.97) for 1–2 drinks/day, 1.08 (95 % CI, 1.01–1.15) for more than 2–4 drinks/day, and 1.14 (95 % CI, 1.02–1.28) for more than 4 drinks/day, with low between-study heterogeneity in all categories (*I* ^2^ ≤ 23.7 %). When the lowest category was split into 3 or less drinks/week and more than 3–7 drinks/week, the overall RRs were 0.89 (95 % CI, 0.84–0.94; *I* ^2^ = 20 %; 16 studies) and 0.90 (95 % CI, 0.83–0.98; *I* ^2^ = 23.6 %; 19 studies), respectively. There were no overall associations of light and moderate alcohol consumption (up to 2 drinks/day) with risk of intracerebral hemorrhage or subarachnoid hemorrhage. However, high alcohol consumption (> 2–4 drinks/day) was associated with a non-statistically significant increased risk of both hemorrhagic stroke subtypes and heavy alcohol consumption (> 4 drinks/day) was associated with a statistically significant increased risk of both intracerebral hemorrhage (RR = 1.67; 95 % CI, 1.25–2.23) and subarachnoid hemorrhage (RR = 1.82; 95 % CI, 1.18–2.82). There was low to moderate heterogeneity among study-specific results for hemorrhagic stroke subtypes. Results did not change in a sensitivity analysis in which the mid-point for the highest category was set at 1.5 times the half range of the preceding category.

#### Subgroup analyses

Results from subgroup analyses by geographic area, sex, and study quality are shown in Table 2. High-to-heavy alcohol consumption was associated with a statistically significant higher risk of ischemic stroke in studies from the United States and Europe but not Asia, but this difference was not statistically significant in meta-regression analysis (United States/Europe vs. Asia: *P* for difference = 0.09). High-to-heavy alcohol consumption appeared to be more strongly positively associated with risk of all stroke types in women than in men but none of the differences were statistically significant (*P* for difference > 0.10). Results for women were similar when light-to-moderate alcohol consumption was defined as 1 or less drinks/day (rather than ≤ 2 drinks/day). The strengths of the positive association between alcohol consumption and subarachnoid hemorrhage varied by geographic area but the number of studies in each stratum was limited and the differences were not statistically significant (*P* for difference > 0.17).

**Table 2:** Relative risks of stroke types for light-to-moderate and high-to-heavy alcohol consumption, overall and stratified by geographic area, sex, and study quality

	Alcohol consumption					
	Light-to-moderate (≤ 2 drinks/day)			High-to-heavy (> 2 drinks/day)		
	No.^a^	RR (95 % CI)	*I* ^2b^	No.^a^	RR (95 % CI)	*I* ^2b^
Ischemic stroke						
All studies	24	0.91 (0.88–0.94)	9.1 %	23	1.09 (1.03–1.16)	0 %
Geographic area						
United States	9	0.90 (0.85–0.94)	7.7 %	8	1.12 (1.01–1.24)	0 %
Europe	9	0.93 (0.88–0.98)	11.1 %	8	1.14 (1.04–1.25)	0 %
Asia	5	0.87 (0.78–0.98)	10.6 %	6	1.07 (0.93–1.23)	30.6 %
Sex						
Men	12	0.94 (0.88–1.00)	18.4 %	10	1.11 (1.00–1.23)	15.2 %
Women	7	0.88 (0.83–0.95)	0 %	6	1.15 (0.96–1.36)	0 %
Women^c^	7	0.88 (0.79–0.98)	32.2 %	7	0.96 (0.85–1.08)	7.9 %
Study quality						
NOS ≥ 7	18	0.90 (0.86–0.93)	0 %	17	1.09 (1.02–1.16)	0 %
NOS < 7	5	0.87 (0.81–0.94)	13.6 %	4	1.10 (0.97–1.25)	9.3 %
Intracerebral hemorrhage						
All studies	11	0.95 (0.84–1.07)	10.2 %	9	1.45 (1.18–1.78)	55.0 %
Geographic area						
United States	3	0.94 (0.75–1.17)	33.4 %	2	1.38 (0.86–2.21)	33.6 %
Europe	4	0.92 (0.77–1.10)	0 %	3	1.39 (0.98–1.96)	40.2 %
Asia	4	1.09 (0.79–1.49)	34.1 %	4	1.56 (1.13–2.17)	69.7 %
Sex						
Men	5	0.98 (0.78–1.24)	26.4 %	5	1.35 (1.06–1.72)	59.4 %
Women	3	0.95 (0.76–1.19)	0 %	2	2.23 (1.47–3.38)	0 %
Women^c^	3	0.81 (0.55–1.19)	37.6 %	3	1.54 (1.08–2.18)	29.6 %
Study quality						
NOS ≥ 7	7	0.99 (0.84–1.18)	7.5 %	6	1.68 (1.32–2.14)	25.4 %
NOS < 7	4	0.90 (0.76–1.07)	17.0 %	3	1.20 (0.92–1.56)	51.9 %
Subarachnoid hemorrhage						
All studies	10	1.16 (0.98–1.37)	0 %	9	1.57 (1.18–2.09)	39.0 %
Geographic area						
United States	3	1.46 (1.02–2.10)	21.4 %	2	2.04 (1.12–3.73)	24.4 %
Europe	4	1.03 (0.81–1.30)	0 %	3	1.17 (0.58–2.37)	58.9 %
Asia	3	1.26 (0.81–1.97)	19.8 %	4	1.57 (1.10–2.24)	31.3 %
Sex						
Men	5	1.06 (0.69–1.60)	29.9 %	5	1.48 (0.96–2.28)	51.0 %
Women	3	1.38 (1.04–1.85)	0 %	2	1.90 (1.16–3.13)	0 %
Women^c^	3	1.56 (1.01–2.38)	19.5 %	3	1.54 (1.08–2.20)	0 %
Study quality						
NOS ≥ 7	7	1.25 (0.97–1.62)	13.4 %	3	1.44 (1.01–2.06)	16.5 %
NOS < 7	4	1.10 (0.87–1.39)	0 %	6	1.57 (1.03–2.40)	50.0 %

*CI* confidence interval, *NOS* Newcastle–Ottawa Scale, *RR* relative risk

^a^Number of studies that contributed data to each category of alcohol consumption and strata

^b^Test for between-study heterogeneity. *I* ^*2*^ values < 30 %, 30–75 %, and > 75 % were interpreted as no or low heterogeneity, moderate heterogeneity, and notable heterogeneity, respectively

^c^Sensitivity analysis in which light-to-moderate alcohol consumption for women was defined as ≤ 1 drink/day and high-to-heavy consumption was defined as > 1 drink/day

The association between alcohol consumption and stroke types did not differ appreciably by reference group used, except that light-to-moderate drinking was not associated with ischemic stroke when occasional drinkers were used as the comparison group (Additional file 1: Table S3).

#### Small-study bias

Indication of small-study bias was observed in the light alcohol consumption category for ischemic stroke (*P* = 0.04) and subarachnoid hemorrhage (*P* = 0.01), but not in the moderate, high, and heavy alcohol consumption categories. There was no evidence of small-study bias for subarachnoid hemorrhage.

## Discussion

Findings from this meta-analysis of 27 prospective studies indicate that alcohol consumption has divergent associations by stroke type. Light and moderate alcohol consumption (up to 2 drinks/day) was associated with a reduced risk of ischemic stroke but was not associated with risk of intracerebral or subarachnoid hemorrhage. Heavy alcohol consumption was associated with an increased risk of all stroke types but with stronger associations for hemorrhagic stroke subtypes than for ischemic stroke.

The divergent dose–response relationships between alcohol consumption and risk of ischemic stroke and hemorrhagic stroke subtypes suggest that different mechanisms underlie associations with the different stroke types. Alcohol consumption is associated with increased high-density lipoprotein cholesterol levels and reduced fibrinogen levels [1–4], and this might explain the lower the risk of ischemic stroke but not hemorrhagic stroke associated with light and moderate alcohol consumption. The adverse impact of alcohol consumption on blood pressure may directly increase the risk of hemorrhagic stroke and outweigh potential beneficial associations of light to moderate drinking with ischemic stroke.

Ischemic stroke itself is caused by a number of different pathophysiological mechanisms and alcohol drinking might have contrasting effects on different ischemic stroke subtypes. For example, moderate and high alcohol consumption is associated with an elevated risk of atrial fibrillation [43], which is a risk factor for cardioembolic stroke. However, only one study in this meta-analysis reported results for ischemic stroke subtypes [24]. Results from that cohort study of Japanese men showed that moderate and heavy alcohol consumption was associated with a non-statistically significant increased risk of cardioembolic stroke, whereas moderate alcohol consumption was associated with a statistically significant reduced risk of lacunar infarction and a non-statistically significant lower risk of large artery stroke [24].

Although the overall evidence from prospective observational studies indicates an inverse association between light-to-moderate alcohol consumption and risk of ischemic stroke, the causality of this association is unclear. Further information on causality can be obtained by looking at genetic associations. This approach, known as Mendelian randomization, avoids some of the crucial limitations of observational studies because allocation of genetic variants is random with regard to potential confounders. A recent Mendelian randomization study shed doubts on the beneficial effects of moderate alcohol consumption on cardiovascular disease. That study showed that individuals with an alcohol dehydrogenase 1B gene variant, which is associated with nondrinking and lower levels of usual alcohol consumption, had a reduced risk of ischemic stroke [44]. However, the gene studied explains only a fraction of alcohol consumption in the population and it may have effects on cardiovascular disease beyond those explained by alcohol consumption. Therefore, it has been suggested that the alcohol dehydrogenase 1B gene allele violates the assumptions required for a variable for Mendelian randomization and is therefore inappropriate for judging the effects of alcohol consumption on cardiovascular disease [45]. To further evaluate the causality of the inverse association between light-to-moderate alcohol consumption and ischemic stroke, additional Mendelian randomization studies that use a better instrumental variable than a single nucleotide polymorphism of the alcohol dehydrogenase 1B gene are required.

A strength of the present meta-analysis is the relatively large number of studies included. Therefore, the association between alcohol consumption and stroke types could be estimated with a relatively high accuracy across a wide range of alcohol consumption, and in different subgroups. In addition, by inclusion of prospective studies, only the possibility that the results may have been affected by recall or selection bias, which could be of concern in case–control studies, was minimized.

This meta-analysis is limited by the lack of individual patient data. We therefore could not adjust the risk estimates for the same covariates in all studies. Some degree of underreporting of alcohol consumption by participants in the included studies is likely to have occurred. Such underreporting would underestimate the threshold for adverse effects of alcohol consumption on stroke risk. Another limitation is that the major type of alcoholic beverage consumed and the drinking pattern differ across populations and this could introduce between-study heterogeneity. Nevertheless, we conducted stratified analyses by geographic area, which in part could account for different alcohol consumption patterns, and found similar associations across regions. Alcohol consumption patterns may be different for populations in southern compared with northern Europe. Because all European studies of alcohol consumption and stroke types were conducted in northern Europe (mainly in the Nordic countries), we could not investigate potential differences in alcohol–stroke type associations for northern versus southern European populations. A further shortcoming is that the association of alcohol consumption with etiologic subtypes of ischemic stroke could not be assessed. Finally, as in any meta-analysis, small-study bias (e.g., publication bias) could be of concern. There was evidence of such bias in the light alcohol consumption category for both ischemic stroke and subarachnoid hemorrhage. Hence, we cannot rule out the possibility that the associations between light alcohol drinking and risk of ischemic stroke and subarachnoid hemorrhage have been overestimated. No indication of small-study bias was observed in the analyses of intracerebral hemorrhage.

Future studies should evaluate the association of alcohol drinking patterns (regular or binge drinking) and usual type of beverage (wine, beer, and liquor) consumed with risk of stroke and ischemic stroke subtypes (large vessel, small vessel, and cardioembolic stroke). Furthermore, future studies should assess underreporting of alcohol intake and may use new approaches for estimating alcohol consumption based on metabolic profiling [46], which offers promise as a way of avoiding errors in self-reporting of alcohol consumption.

## Conclusions

Findings from this meta-analysis indicate that alcohol consumption has divergent effects on different stroke types. This may explain some of the inconsistent results from previous studies associating alcohol consummation with all strokes.

## Additional file

Additional file 1:
**Text S1.** Methods for the Cohort of Swedish Men and Swedish Mammography Cohort. **Table S1.** Age-standardized baseline characteristics by alcohol consumption in the Cohort of Swedish Men and Swedish Mammography Cohort. **Table S2.** Relative risks of ischemic stroke, intracerebral hemorrhage, and subarachnoid hemorrhage according to alcohol consumption in the Cohort of Swedish Men and the Swedish Mammography Cohort. **Table S3.** Relative risks of stroke types for light-to-moderate and high-to-heavy alcohol consumption, stratified by alcohol intake reference group. **Figure S1.** Newcastle–Ottawa Scale for prospective cohort studies: Details of how the criteria were applied for prospective studies of the association of alcohol consumption with stroke. **Figure S2.** Flow chart of study selection (DOCX 202 kb)

## Abbreviations

CI: confidence interval
COSM: Cohort of Swedish Men
RR: relative risk
SMC: Swedish Mammography Cohort

## References

[1] Mukamal KJ, Rimm EB (2008). Alcohol consumption: risks and benefits. Curr Atheroscler Rep.

[2] O’Keefe JH, Bybee KA, Lavie CJ (2007). Alcohol and cardiovascular health: the razor-sharp double-edged sword. J Am Coll Cardiol.

[3] Agarwal DP (2002). Cardioprotective effects of light-moderate consumption of alcohol: a review of putative mechanisms. Alcohol Alcohol.

[4] Brien SE, Ronksley PE, Turner BJ, Mukamal KJ, Ghali WA (2011). Effect of alcohol consumption on biological markers associated with risk of coronary heart disease: systematic review and meta-analysis of interventional studies. BMJ.

[5] Taylor B, Irving HM, Baliunas D, Roerecke M, Patra J, Mohapatra S, Rehm J (2009). Alcohol and hypertension: gender differences in dose-response relationships determined through systematic review and meta-analysis. Addiction.

[6] Ronksley PE, Brien SE, Turner BJ, Mukamal KJ, Ghali WA (2011). Association of alcohol consumption with selected cardiovascular disease outcomes: a systematic review and meta-analysis. BMJ.

[7] Roerecke M, Rehm J (2012). The cardioprotective association of average alcohol consumption and ischaemic heart disease: a systematic review and meta-analysis. Addiction.

[8] Reynolds K, Lewis B, Nolen JD, Kinney GL, Sathya B, He J (2003). Alcohol consumption and risk of stroke: a meta-analysis. JAMA.

[9] Patra J, Taylor B, Irving H, Roerecke M, Baliunas D, Mohapatra S, Rehm J (2010). Alcohol consumption and the risk of morbidity and mortality for different stroke types--a systematic review and meta-analysis. BMC Public Health.

[10] Stroup DF, Berlin JA, Morton SC, Olkin I, Williamson GD, Rennie D, Moher D, Becker BJ, Sipe TA, Thacker SB (2000). Meta-analysis of observational studies in epidemiology: a proposal for reporting. Meta-analysis Of Observational Studies in Epidemiology (MOOSE) group. JAMA.

[11] The Newcastle-Ottawa Scale (NOS) for assessing the quality of nonrandomised studies in meta-analyses. http://www.ohri.ca/programs/clinical_epidemiology/oxford.asp. Accessed 1 Sept 2016.

[12] Higgins JP, Thompson SG (2002). Quantifying heterogeneity in a meta-analysis. Stat Med.

[13] Egger M, Davey Smith G, Schneider M, Minder C (1997). Bias in meta-analysis detected by a simple, graphical test. BMJ.

[14] Donahue RP, Abbott RD, Reed DM, Yano K (1986). Alcohol and hemorrhagic stroke. The Honolulu Heart Program. JAMA.

[15] Stampfer MJ, Colditz GA, Willett WC, Speizer FE, Hennekens CH (1988). A prospective study of moderate alcohol consumption and the risk of coronary disease and stroke in women. N Engl J Med.

[16] Iso H, Kitamura A, Shimamoto T, Sankai T, Naito Y, Sato S, Kiyama M, Iida M, Komachi Y (1995). Alcohol intake and the risk of cardiovascular disease in middle-aged Japanese men. Stroke.

[17] Kiyohara Y, Kato I, Iwamoto H, Nakayama K, Fujishima M (1995). The impact of alcohol and hypertension on stroke incidence in a general Japanese population. The Hisayama Study. Stroke.

[18] Leppala JM, Paunio M, Virtamo J, Fogelholm R, Albanes D, Taylor PR, Heinonen OP (1999). Alcohol consumption and stroke incidence in male smokers. Circulation.

[19] Sankai T, Iso H, Shimamoto T, Kitamura A, Naito Y, Sato S, Okamura T, Imano H, Iida M, Komachi Y (2000). Prospective study on alcohol intake and risk of subarachnoid hemorrhage among Japanese men and women. Alcohol Clin Exp Res.

[20] Suh I, Jee SH, Kim HC, Nam CM, Kim IS, Appel LJ (2001). Low serum cholesterol and haemorrhagic stroke in men: Korea Medical Insurance Corporation Study. Lancet.

[21] Klatsky AL, Armstrong MA, Friedman GD, Sidney S (2001). Alcohol drinking and risk of hospitalization for ischemic stroke. Am J Cardiol.

[22] Klatsky AL, Armstrong MA, Friedman GD, Sidney S (2002). Alcohol drinking and risk of hemorrhagic stroke. Neuroepidemiology.

[23] Djousse L, Ellison RC, Beiser A, Scaramucci A, D’Agostino RB, Wolf PA (2002). Alcohol consumption and risk of ischemic stroke: The Framingham Study. Stroke.

[24] Iso H, Baba S, Mannami T, Sasaki S, Okada K, Konishi M, Tsugane S, JPHC Study Group (2004). Alcohol consumption and risk of stroke among middle-aged men: the JPHC Study Cohort I. Stroke.

[25] Iwashita M, Matsushita Y, Sasaki J, Arakawa K, Kono S, Kyushu Lipid Intervention Study Group (2005). Relation of serum total cholesterol and other factors to risk of cerebral infarction in Japanese men with hypercholesterolemia. Circ J.

[26] Mukamal KJ, Chung H, Jenny NS, Kuller LH, Longstreth WT, Mittleman MA, Burke GL, Cushman M, Beauchamp NJ, Siscovick DS (2005). Alcohol use and risk of ischemic stroke among older adults: the cardiovascular health study. Stroke.

[27] Nielsen NR, Truelsen T, Barefoot JC, Johnsen SP, Overvad K, Boysen G, Schnohr P, Gronbaek M (2005). Is the effect of alcohol on risk of stroke confined to highly stressed persons?. Neuroepidemiology.

[28] Elkind MS, Sciacca R, Boden-Albala B, Rundek T, Paik MC, Sacco RL (2006). Moderate alcohol consumption reduces risk of ischemic stroke: the Northern Manhattan Study. Stroke.

[29] Bazzano LA, Gu D, Reynolds K, Wu X, Chen CS, Duan X, Chen J, Wildman RP, Klag MJ, He J (2007). Alcohol consumption and risk for stroke among Chinese men. Ann Neurol.

[30] Sturgeon JD, Folsom AR, Longstreth WT, Shahar E, Rosamond WD, Cushman M (2007). Risk factors for intracerebral hemorrhage in a pooled prospective study. Stroke.

[31] Chiuve SE, Rexrode KM, Spiegelman D, Logroscino G, Manson JE, Rimm EB (2008). Primary prevention of stroke by healthy lifestyle. Circulation.

[32] Lu M, Ye W, Adami HO, Weiderpass E (2008). Stroke incidence in women under 60 years of age related to alcohol intake and smoking habit. Cerebrovasc Dis.

[33] Sandvei MS, Romundstad PR, Muller TB, Vatten L, Vik A (2009). Risk factors for aneurysmal subarachnoid hemorrhage in a prospective population study: the HUNT study in Norway. Stroke.

[34] Bos S, Grobbee DE, Boer JM, Verschuren WM, Beulens JW (2010). Alcohol consumption and risk of cardiovascular disease among hypertensive women. Eur J Cardiovasc Prev Rehabil.

[35] Rist PM, Berger K, Buring JE, Kase CS, Gaziano JM, Kurth T (2010). Alcohol consumption and functional outcome after stroke in men. Stroke.

[36] Zhang Y, Tuomilehto J, Jousilahti P, Wang Y, Antikainen R, Hu G (2011). Lifestyle factors on the risks of ischemic and hemorrhagic stroke. Arch Intern Med.

[37] Higashiyama A, Wakabayashi I, Ono Y, Watanabe M, Kokubo Y, Okayama A, Miyamoto Y, Okamura T (2011). Association with serum gamma-glutamyltransferase levels and alcohol consumption on stroke and coronary artery disease: the Suita study. Stroke.

[38] Drogan D, Sheldrick AJ, Schutze M, Knuppel S, Andersohn F, di Giuseppe R, Herrmann B, Willich SN, Garbe E, Bergmann MM (2012). Alcohol consumption, genetic variants in alcohol deydrogenases, and risk of cardiovascular diseases: a prospective study and meta-analysis. PLoS One.

[39] Jimenez M, Chiuve SE, Glynn RJ, Stampfer MJ, Camargo CA, Willett WC, Manson JE, Rexrode KM (2012). Alcohol consumption and risk of stroke in women. Stroke.

[40] Ikehara S, Iso H, Yamagishi K, Kokubo Y, Saito I, Yatsuya H, Inoue M, Tsugane S, Group JS (2013). Alcohol consumption and risk of stroke and coronary heart disease among Japanese women: the Japan Public Health Center-based prospective study. Prev Med.

[41] Kadlecova P, Andel R, Mikulik R, Handing EP, Pedersen NL (2015). Alcohol consumption at midlife and risk of stroke during 43 years of follow-up: cohort and twin analyses. Stroke.

[42] Jones SB, Loehr L, Avery CL, Gottesman RF, Wruck L, Shahar E, Rosamond WD (2015). Midlife alcohol consumption and the risk of stroke in the atherosclerosis risk in communities study. Stroke.

[43] Larsson SC, Drca N, Wolk A (2014). Alcohol consumption and risk of atrial fibrillation: a prospective study and dose-response meta-analysis. J Am Coll Cardiol.

[44] Holmes MV, Dale CE, Zuccolo L, Silverwood RJ, Guo Y, Ye Z, Prieto-Merino D, Dehghan A, Trompet S, Wong A (2014). Association between alcohol and cardiovascular disease: Mendelian randomisation analysis based on individual participant data. BMJ.

[45] Critique 143: A Mendelian randomization assessment of alcohol and cardiovascular disease – 20 July 2014. http://www.bu.edu/alcohol-forum/critique-143. Accessed 1 Sept 2016.

[46] Wurtz P, Cook S, Wang Q, Tiainen M, Tynkkynen T, Kangas AJ, Soininen P, Laitinen J, Viikari J, Kahonen M, et al. Metabolic profiling of alcohol consumption in 9778 young adults. Int J Epidemiol. 2016. Ahead of print. 10.1093/ije/dyw175 PMC5100616 27494945

