# Supplementary material for: Differing association of alcohol consumption with different stroke types: a systematic review and meta-analysis
Source: BMC Med. 2016 Nov 24;14:178. doi: 10.1186/s12916-016-0721-4 (PMC5121939; doi:10.1186/s12916-016-0721-4)
Supplement: Additional file 1: — Text S1. Methods for the Cohort of Swedish Men and Swedish Mammography Cohort. Table S1. Age-standardized baseline characteristics by alcohol consumption in the Cohort of Swedish Men and Swedish Mammography Cohort. Table S2. Relative risks of ischemic stroke, intracerebral hemorrhage, and subarachnoid hemorrhage according to alcohol consumption in the Cohort of Swedish Men and the Swedish Mammography Cohort. Table S3. Relative risks of stroke types for light-to-moderate and high-to-heavy alcohol consumption, stratified by alcohol intake reference group. Figure S1. Newcastle–Ottawa Scale for prospective cohort studies: Details of how the criteria were applied for prospective studies of the association of alcohol consumption with stroke. Figure S2. Flow chart of study selection (DOCX 202 kb) [file 12916_2016_721_MOESM1_ESM.docx]

**SUPPLEMENTARY MATERIAL**

**Differing association of alcohol consumption with different stroke types: a systematic review and meta-analysis**

Susanna C. Larsson, Alice Wallin, Alicja Wolk, Hugh S. Markus

**CONTENT**

**Text S1:** Methods for the Cohort of Swedish Men and Swedish Mammography Cohort 2

**Table S1:** Age-standardized baseline characteristics by alcohol consumption in the Cohort of Swedish Men and Swedish Mammography Cohort 4

**Table S2:** Relative risks of ischemic stroke, intracerebral hemorrhage, and subarachnoid hemorrhage according to alcohol consumption in the Cohort of Swedish Men and the Swedish Mammography Cohort 5

**Table S3:** Relative risks of stroke types for light-to-moderate and high-to-heavy alcohol consumption, stratified by alcohol intake reference group ... 7

**Figure S1:** Newcastle-Ottawa Scale for prospective cohort studies: Details of how the criteria were applied for prospective studies of the association of alcohol consumption with stroke.8

**Figure S2:** Flow chart of study selection ........………………………………………………………………………9

# Text S1: Methods

**Cohort of Swedish Men and Swedish Mammography Cohort**

This study included participants of the population-based Cohort of Swedish Men and the Swedish Mammography Cohort. These cohorts have been described in detail previously.[^1^](#_ENREF_1) Briefly, during the late autumn of 1997, 48 850 men (45–79 years of age) and 39 227 women (49–83 years of age) from three Swedish counties completed a questionnaire about diet, alcohol consumption, and other potential risk factor for chronic diseases. We excluded participants with an erroneous or a missing personal identification number (n=540), those who died before start of follow-up (n=81), those with a diagnosis in the Swedish registries of stroke or ischemic heart disease (n=7790) or cancer (n=4403) before baseline, and those who did not provide data on alcohol consumption (n=1676), leaving 73 587 participants (39 941 men and 33 646 women) for analysis. The Regional Ethical Review Board at Karolinska Institutet in Stockholm, Sweden, approved the study. Completion of the questionnaire was considered to imply informed consent.

**Assessment of alcohol intake**

A food-frequency questionnaire with 96 foods/food items and beverages, including six alcoholic beverages, was used to assess alcohol consumption during the past year. Participants were asked if they have never had alcohol or if they had stopped drinking alcohol. Current drinkers reported they usual consumption of class I beer (alcohol by volume, <2.25%), class II beer (2.8−3.5%), class III beer (>3.5%), wine (12%), strong wine (>18%), and liquor as well as the amount of beer, wine, and liquor consumed on a single occasion (open question without predefined categories). For frequency of consumption of alcoholic beverages, there were nine predefined categories, ranging from never to ≥3 times per day. Weekly alcohol consumption was computed by multiplying the frequency of consumption of each alcoholic beverage by the amount consumed. One drink was assumed to correspond to 12 g alcohol (one standard drink in Sweden). This amount corresponds to about 660 ml class I beer, 500 ml class II beer, 330 ml class III beer, 150 ml wine, 80 ml strong wine or 40 ml liquor. In a validation study of the questionnaire, the correlation coefficient between estimates from the questionnaire and the mean of fourteen 24-hour recall interviews was 0.81 for alcohol (ethanol).[^2^](#_ENREF_2)

**Assessment of covariates**

The baseline questionnaire collected information on education, family history of myocardial infarction before the age of 60 years, smoking, weight, height, physical activity (including walking/bicycling and exercise), aspirin use, and history of hypertension, high cholesterol levels and diabetes. Self-reported history of hypertension was complemented with information on diagnosis of hypertension in the Swedish National Patient Register. Diabetes was defined as self-reported diabetes in the questionnaire or a diagnosis in the Swedish National Patient or Diabetes Registers. Data on atrial fibrillation was acquired from the Swedish National Patient Register. Body mass index was derived from weight divided by height squared (kg/m^2^).

**Case ascertainment and follow-up**

Cases of stroke were identified by record linkage, using the Swedish Personal Identiﬁcation number assigned to each Swedish resident, to the Swedish National Patient Register (includes both inpatient and outpatient [non-primary care]) and the Swedish Cause of Death Register. The International Classification of Diseases 10th Revision was used to classify the strokes as ischemic stroke (code I63), intracerebral hemorrhage (I61), and subarachnoid hemorrhage (I60). Validation studies have shown that 96% of stroke cases are identified via the Swedish National Patient Register[^3^](#_ENREF_3) and 92% of the cases are correctly classified.[^4^](#_ENREF_4) Dates of deaths were ascertained through linkage with the Swedish Cause of Death Register, which is 100% complete. Participants were followed up from January 1, 1998 to the date of diagnosis of first stroke, date of death or December 31, 2010, whichever was sooner.

**Statistical analysis**

Participants were categorized into seven groups according to their alcohol drinking status and number of drinks consumed per week: never drinkers (lifetime abstainers), former drinkers, and current drinkers of <1 (reference group), 1–6, 7–14, 15–21, and >21 drinks per week. Cox proportional hazards regression models, with age as the time scale, was used to compute hazard ratios (hereafter referred to as relative risks [RR]) with 95% confidence intervals (CI). Analyses for men and women combined (pooled analysis of the two cohorts) were stratified by sex to allow the baseline hazard to differ for men and women. Multivariable models included adjustment for education (less than high school, high school, or university), family history of myocardial infarction before the age of 60 years (yes/no), smoking (never, past, or current smoker), body mass index (kg/m^2^; <25, 25–29.9, or ≥30), walking/bicycling (four categories), exercise (four categories), aspirin use (nonuser, <7 tablets/week, or ≥7 tablets/week), and history of hypertension (yes/no), hypercholesterolemia (yes/no), diabetes (yes/no) and atrial fibrillation (yes/no). The multivariable model was further controlled for a modified Dietary Approaches to Stop Hypertension diet score (in quartiles), which was recently reported to be associated with the risk of stroke in this study population.[^5^](#_ENREF_5) The proportional hazards assumption was tested using Schoenfeld residuals, and no violation of the assumption was found.

Tests for trend across categories of alcohol consumption in current drinkers were performed by generating a variable containing the median value for each alcohol consumption category and modeling this variable as a continuous variable in the Cox model. The statistical analyses were performed using SAS (version 9.4, SAS Institute, Cary, NC). All statistical tests were 2-sided (α=0.05).

**References**

**1.** Larsson SC, Åkesson A, Wolk A. Egg consumption and risk of heart failure, myocardial infarction, and stroke: results from 2 prospective cohorts. *Am J Clin Nutr.* 2015;102(5):1007-1013.

**2.** Messerer M, Johansson SE, Wolk A. The validity of questionnaire-based micronutrient intake estimates is increased by including dietary supplement use in Swedish men. *J Nutr.* 2004;134(7):1800-1805.

**3.** Ludvigsson JF, Andersson E, Ekbom A, et al. External review and validation of the Swedish national inpatient register. *BMC Public Health.* 2011;11:450.

**4.** Appelros P, Terent A. Validation of the Swedish inpatient and cause-of-death registers in the context of stroke. *Acta Neurol Scand.* 2011;123(4):289-293.

**5.** Larsson SC, Wallin A, Wolk A. Dietary Approaches to Stop Hypertension Diet and Incidence of Stroke: Results From 2 Prospective Cohorts. *Stroke.* 2016;47(4):986-990.

## Table S1: Age-standardized baseline characteristics by alcohol consumption in the Cohort of Swedish Men and Swedish Mammography Cohort

|  | **Alcohol consumption** | | | | | | | | | | | | | | |
| --- | --- | --- | --- | --- | --- | --- | --- | --- | --- | --- | --- | --- | --- | --- | --- |
|  | **Cohort of Swedish Men (*n* = 39 941 men)** | | | | | | |  | **Swedish Mammography Cohort (*n* = 33 646 women)** | | | | | | |
|  |  |  | **Current, drinks/week** | | | | |  |  |  | **Current, drinks/week** | | | | |
| **Characteristics** | **Never*** | **Former** | **<1** | **1–6** | **7–14** | **15–21** | **>21** |  | **Never*** | **Former** | **<1** | **1–6** | **7–14** | **15–21** | **>21** |
| Participants, *n* | 1790 | 2266 | 3485 | 16 172 | 9885 | 3552 | 2791 |  | 4037 | 879 | 7984 | 16 240 | 3609 | 606 | 291 |
| Age, years, mean | 64.1 | 63.5 | 61.9 | 59.7 | 57.6 | 56.6 | 56.4 |  | 67.5 | 62.1 | 63.2 | 59.6 | 57.5 | 57.0 | 58.0 |
| Postsecondary education, % | 16.0 | 11.8 | 15.0 | 16.8 | 20.0 | 20.0 | 19.1 |  | 12.4 | 14.0 | 15.1 | 21.7 | 28.3 | 35.3 | 29.9 |
| Family history of MI, % | 11.8 | 15.6 | 14.5 | 14.1 | 14.2 | 14.2 | 15.7 |  | 16.7 | 18.8 | 17.2 | 16.4 | 16.1 | 16.8 | 18.6 |
| Current smokers, % | 6.3 | 32.1 | 20.8 | 23.2 | 25.2 | 30.0 | 38.4 |  | 10.2 | 37.7 | 21.4 | 24.6 | 31.1 | 36.7 | 38.8 |
| Overweight, % | 52.5 | 58.5 | 53.8 | 54.1 | 54.4 | 58.8 | 60.2 |  | 52.4 | 48.0 | 47.6 | 40.5 | 38.6 | 37.9 | 43.1 |
| Walk/bicycle ≥40 min/day, % | 33.2 | 37.5 | 33.9 | 32.9 | 31.9 | 32.1 | 30.0 |  | 36.0 | 35.8 | 37.7 | 36.1 | 36.0 | 36.1 | 31.8 |
| Exercise ≥2 hours/week, % | 53.7 | 58.6 | 56.6 | 58.7 | 60.5 | 57.9 | 56.2 |  | 52.9 | 53.4 | 55.7 | 58.9 | 57.6 | 56.6 | 48.9 |
| Aspirin use ≥7 tablets/week, % | 3.7 | 8.0 | 5.1 | 5.2 | 4.9 | 6.0 | 6.9 |  | 9.0 | 11.8 | 8.9 | 7.9 | 9.4 | 8.3 | 9.6 |
| Hypertension, % | 19.3 | 24.2 | 22.3 | 21.1 | 21.0 | 22.2 | 25.9 |  | 21.8 | 24.9 | 21.1 | 18.9 | 18.1 | 15.7 | 19.5 |
| Hypercholesterolemia, % | 9.1 | 15.0 | 12.2 | 12.7 | 13.2 | 15.2 | 15.9 |  | 7.7 | 7.6 | 8.2 | 7.5 | 6.6 | 7.6 | 6.8 |
| Diabetes, % | 8.9 | 12.7 | 8.8 | 7.4 | 6.4 | 6.3 | 7.9 |  | 5.8 | 7.7 | 4.8 | 2.7 | 2.3 | 1.6 | 5.2 |
| Atrial fibrillation, % | 1.8 | 3.3 | 2.0 | 2.0 | 2.2 | 2.3 | 2.4 |  | 1.2 | 2.4 | 1.1 | 1.1 | 1.0 | 1.2 | 1.3 |
| mDASH diet score, mean† | 20.6 | 20.3 | 20.3 | 20.5 | 20.9 | 20.7 | 20.1 |  | 22.1 | 21.8 | 22.5 | 22.9 | 22.7 | 22.2 | 21.8 |

Abbreviations: BMI = body mass index; mDASH = modified Dietary Approaches to Stop Hypertension; MI = myocardial infarction.

*Lifetime abstainers.

†A measure of a healthy diet; the mDASH score ranges from 7 (minimal adherence) to 35 (maximal adherence).

### Table S2: Relative risks of ischemic stroke, intracerebral hemorrhage, and subarachnoid hemorrhage according to alcohol consumption in the Cohort of Swedish Men and the Swedish Mammography Cohort

|  | **Ischemic stroke** | | | |  | **Intracerebral hemorrhage** | | |  | **Subarachnoid hemorrhage** | | |
| --- | --- | --- | --- | --- | --- | --- | --- | --- | --- | --- | --- | --- |
| **Alcohol consumption** | **Cases** | **Person-years*** | **Age-adjusted**  **RR (95% CI)** | **Multivariable**  **RR (95% CI)†** |  | **Cases** | **Age-adjusted**  **RR (95% CI)** | **Multivariable**  **RR (95% CI)†** |  | **Cases** | **Age-adjusted**  **RR (95% CI)** | **Multivariable**  **RR (95% CI)†** |
| **COSM (*n* = 39 941)** |  |  |  |  |  |  |  |  |  |  |  |  |
| Drinking status |  |  |  |  |  |  |  |  |  |  |  |  |
| Never | 145 | 20 022 | 1.10 (0.89–1.35) | 1.12 (0.91–1.39) |  | 28 | 1.45 (0.88–2.40) | 1.50 (0.90–2.49) |  | 2 | 0.38 (0.08–1.75) | 0.41 (0.09–1.87) |
| Former | 171 | 23 919 | 1.16 (0.95–2.42) | 1.09 (0.89–2.33) |  | 33 | 1.53 (0.95–2.48) | 1.51 (0.93–2.46) |  | 4 | 0.65 (0.21–2.09) | 0.62 (0.19–1.98) |
| Current, drinks/wk‡ |  |  |  |  |  |  |  |  |  |  |  |  |
| <1 (0.6)§ | 221 | 39 599 | 1.00 (reference) | 1.00 (reference) |  | 33 | 1.00 (reference) | 1.00 (reference) |  | 10 | 1.00 (reference) | 1.00 (reference) |
| 1–6 (3.8) | 923 | 189 863 | 1.04 (0.90–1.20) | 1.05 (0.91–1.22) |  | 134 | 0.99 (0.67–1.44) | 0.98 (0.67–1.44) |  | 33 | 0.70 (0.35–1.43) | 0.70 (0.34–1.42) |
| 7–14 (9.8) | 464 | 118 905 | 1.01 (0.86–1.19) | 1.04 (0.89–1.23) |  | 63 | 0.87 (0.57–1.33) | 0.87 (0.57–1.34) |  | 26 | 0.90 (0.43–1.89) | 0.89 (0.42–1.87) |
| 15–21 (16.6) | 159 | 42 739 | 1.08 (0.88–1.33) | 1.08 (0.88–1.33) |  | 28 | 1.19 (0.72–1.97) | 1.19 (0.71–1.99) |  | 3 | 0.30 (0.08–1.08) | 0.29 (0.08–1.05) |
| >21 (28.7) | 133 | 32 844 | 1.21 (0.98–1.51) | 1.15 (0.93–1.44) |  | 31 | 1.76 (1.07–2.88) | 1.68 (1.02–1.78) |  | 4 | 0.52 (0.16–1.66) | 0.45 (0.14–1.48) |
| *P* for trend\|\| |  |  | 0.12 | 0.35 |  |  | 0.008 | 0.02 |  |  | 0.22 | 0.14 |
| **SMC (*n* = 33 646)** |  |  |  |  |  |  |  |  |  |  |  |  |
| Drinking status |  |  |  |  |  |  |  |  |  |  |  |  |
| Never | 319 | 45 429 | 1.00 (0.86–1.15) | 0.99 (0.86–1.14) |  | 32 | 0.97 (0.62–1.51) | 1.08 (0.69–1.70) |  | 10 | 0.98 (0.46–2.08) | 1.02 (0.39–1.82) |
| Former | 39 | 10 037 | 0.88 (0.64–1.22) | 0.77 (0.55–1.07) |  | 5 | 1.02 (0.41–2.56) | 0.79 (0.32–2.00) |  | 0 | – | – |
| Current, drinks/wk‡ |  |  |  |  |  |  |  |  |  |  |  |  |
| <1 (0.4)§ | 477 | 94 735 | 1.00 (reference) | 1.00 (reference) |  | 52 | 1.00 (reference) | 1.00 (reference) |  | 21 | 1.00 (reference) | 1.00 (reference) |
| 1–6 (3.0) | 643 | 199 475 | 0.92 (0.82–1.04) | 0.96 (0.85–1.09) |  | 91 | 1.13 (0.80–1.59) | 1.09 (0.77–1.55) |  | 45 | 1.05 (0.62–1.77) | 1.03 (0.61–1.74) |
| 7–14 (9.2) | 100 | 44 940 | 0.82 (0.66–1.02) | 0.86 (0.69–1.08) |  | 18 | 1.17 (0.68–2.02) | 1.06 (0.61–1.85) |  | 11 | 1.17 (0.56–2.45) | 1.12 (0.53–2.37) |
| 15–21 (16.4) | 19 | 7458 | 1.03 (0.65–1.63) | 1.07 (0.67–1.70) |  | 3 | 1.30 (0.40–4.19) | 1.18 (0.36–3.82) |  | 5 | 3.23 (1.21–8.64) | 3.08 (1.14–8.36) |
| >21 (26.0) | 11 | 3475 | 1.08 (0.59–1.97) | 1.09 (0.60–1.99) |  | 4 | 3.30 (1.19–9.15) | 2.87 (1.03–8.03) |  | 2 | 2.74 (0.64-11.72) | 2.62(0.61-11.33) |
| *P* for trend\|\| |  |  | 0.42 | 0.69 |  |  | 0.06 | 0.13 |  |  | 0.02 | 0.03 |
|  |  |  |  |  |  |  |  |  |  |  |  |  |
| **Pooled analysis**¶ |  |  |  |  |  |  |  |  |  |  |  |  |
| Drinking status |  |  |  |  |  |  |  |  |  |  |  |  |
| Never | 464 | 65 451 | 1.03 (0.91–1.16) | 1.03 (0.92–1.16) |  | 60 | 1.15 (0.83–1.61) | 1.21 (0.86–1.69) |  | 12 | 0.78 (0.40–1.53) | 0.82 (0.42–1.61) |
| Former | 210 | 33 956 | 1.05 (0.90–1.23) | 0.97 (0.83–1.14) |  | 38 | 1.46 (0.99–2.16) | 1.36 (0.92–2.02) |  | 4 | 0.57 (0.20–1.64) | 0.53 (0.19–1.53) |
| Current, drinks/wk‡ |  |  |  |  |  |  |  |  |  |  |  |  |
| <1 (0.4)§ | 698 | 134 334 | 1.00 (reference) | 1.00 (reference) |  | 85 | 1.00 (reference) | 1.00 (reference) |  | 31 | 1.00 (reference) | 1.00 (reference) |
| 1–6 (3.4) | 1566 | 389 338 | 0.96 (0.88–1.05) | 0.99 (0.90–1.08) |  | 225 | 1.04 (0.81–1.35) | 1.03 (0.79–1.33) |  | 78 | 0.94 (0.62–1.43) | 0.93 (0.61–1.43) |
| 7–14 (9.6) | 564 | 163 845 | 0.93 (0.82–1.04) | 0.96 (0.85–1.08) |  | 81 | 0.94 (0.68–1.29) | 0.91 (0.66–1.25) |  | 37 | 1.16 (0.70–1.92) | 1.14 (0.68–1.90) |
| 15–21 (16.6) | 178 | 50 197 | 1.01 (0.85–1.20) | 1.02 (0.86–1.21) |  | 31 | 1.23 (0.80–1.88) | 1.18 (0.76–1.81) |  | 8 | 0.87 (0.39–1.94) | 0.83 (0.37–1.87) |
| >21 (28.4) | 144 | 36 319 | 1.13 (0.94–1.36) | 1.08 (0.89–1.30) |  | 35 | 1.91 (1.26–2.88) | 1.74 (1.15–1.65) |  | 6 | 0.92 (0.37–2.27) | 0.83 (0.33–2.06) |
| *P* for trend\|\| |  |  | 0.28 | 0.54 |  |  | 0.002 | 0.006 |  |  | 0.93 | 0.84 |

Abbreviations: CI = confidence interval; COSM = cohort of Swedish Men; RR = relative risk; SMC = Swedish Mammography Cohort.

*The number of person-years across categories of alcohol consumption is the same for all stroke types.

†Adjusted for age (as the time scale in the Cox model), education, family history of myocardial infarction before 60 years of age, smoking, body mass index, walking/bicycling, exercise, aspirin use, hypertension, hypercholesterolemia, diabetes, atrial fibrillation, and modified Dietary Approaches to Stop Hypertension diet score.

‡Standard drink = 12 g alcohol. One standard drink corresponds to about 330 ml strong beer, 150 ml wine, 80 ml strong wine, and 40 ml liquor.

§The value in parenthesis is the median alcohol consumption in the category.

||*P* for trend across categories of alcohol consumption in current drinkers.

¶The pooled analysis of the two cohorts was further adjusted for sex (cohort) through stratification in the Cox model.

### Table S3: Relative risks of stroke types for light-to-moderate and high-to-heavy alcohol consumption, stratified by alcohol intake reference group

|  |  | **Alcohol consumption** | | | | | | |
| --- | --- | --- | --- | --- | --- | --- | --- | --- |
|  |  | **Light-to-moderate (≤2 drinks/day)** | | |  | **High-to-heavy (>2 drinks/day)** | | |
|  |  | **No.*** | **RR (95% CI)** | ***I*^2^†** |  | **No.*** | **RR (95% CI)** | ***I*^2^†** |
| **Ischemic stroke** | |  |  |  |  |  |  |  |
|  | Nondrinkers‡ | 8 | 0.87 (0.81-0.92) | 0% |  | 6 | 1.13 (0.98-1.29) | 48.3% |
|  | Never drinkers | 8 | 0.87 (0.82-0.91) | 0% |  | 8 | 1.06 (0.95-1.19) | 0% |
|  | Occasional drinkers | 8 | 0.98 (0.94-1.04) | 1.7% |  | 8 | 1.13 (1.03-1.24) | 0% |
| **Intracerebral hemorrhage** | | |  |  |  |  |  |  |
|  | Nondrinkers‡ | 5 | 0.91 (0.64-1.29) | 47.6% |  | 4 | 1.21 (0.87-1.67) | 54.9% |
|  | Never drinkers | 1 | – | – |  | 1 | – | – |
|  | Occasional drinkers | 4 | 1.04 (0.89-1.21) | 0% |  | 4 | 1.74 (1.45-2.09) | 0% |
| **Subarachnoid hemorrhage** | | |  |  |  |  |  |  |
|  | Nondrinkers‡ | 5 | 1.39 (1.00-1.92) | 18.2% |  | 3 | 1.43 (1.00-2.05) | 17.7% |
|  | Never drinkers | 1 | – | – |  | 2 | 1.59 (0.88-2.90) | 44.0% |
|  | Occasional drinkers | 4 | 1.10 (0.84-1.44) | 0% |  | 4 | 1.62 (0.89-2.92) | 55.0% |

Abbreviations: CI = confidence interval; RR = relative risk.

*Number of studies that contributed data to each category of alcohol consumption and strata.

†Test for between-study heterogeneity. *I*^2^ values <30%, 30%–75%, and >75% were interpreted as respectively no or low heterogeneity, moderate heterogeneity, and notable heterogeneity.

‡Former drinkers included.

#### Figure S1: Newcastle-Ottawa Scale for prospective cohort studies: Details of how the criteria were applied for prospective studies of the association of alcohol consumption with stroke

**Selection**

*1) Representativeness of the exposed cohort*

- Star assigned if exposed cohort was truly or somewhat representative of the average alcohol-consumers in the community (i.e., the sample was random or covered all individuals residing in one or a few geographical areas).

*2) Selection of the non-exposed cohort*

- Star assigned where non-exposed participants were drawn from the same population as the exposed participants.

*3) Assessment of exposure*

- Star assigned if alcohol consumption had been assessed using a structured interview.

*4) Demonstration that outcome of interest was not present at the start of study*

- Star assigned to studies that excluded participants with stroke at baseline.

**Comparability**

*1) Comparability of cohorts on the basis of the design or analysis*

- One star assigned to studies that adjusted for age, sex (if applicable), and smoking.
- Second star assigned to studies that further adjusted for body mass index and diabetes (or fasting blood glucose).

**Outcome**

*1) Assessment of outcome*

- Star assigned where outcomes were identified by medical records or record linkage to registers.

*2) Was follow-up long enough for outcomes to occur*

- Star assigned where follow-up was at least 5 years.

*3) Adequacy of follow up of cohorts*

- Star assigned where the loss to follow-up had been estimated and reported in the article, and where loss was no more than 10%.

##### Figure S2: Flow chart of study selection

*The 29 articles reported results from 27 prospective studies. The number of studies on various stroke types does not add up to the total number of articles because some studies reported results on more than one stroke type.
